# Adaptive transfer alignment method based on the observability analysis for airborne pod strapdown inertial navigation system

**DOI:** 10.1038/s41598-021-04732-4

**Published:** 2022-01-18

**Authors:** Weina Chen, Zhong Yang, Shanshan Gu, Yizhi Wang, Yujuan Tang

**Affiliations:** College of Intelligent Science and Control Engineering, Jinling Institute of Technology, Nanjing, China

**Keywords:** Engineering, Physics

## Abstract

For the airborne pod strapdown inertial navigation system, it is necessary to use the host aircraft's inertial navigation system for the transfer alignment as quickly and accurately as possible in the flight process of the aircraft. The purpose of this paper is to propose an adaptive transfer alignment method based on the observability analysis for the strapdown inertial navigation system, which is able to meet the practical need of maintaining the navigation accuracy of the airborne pod. The observability of each state variable is obtained by observability analysis of system state variables. According to the weight of the observability, a transfer alignment filter algorithm based on adaptive adjustment factor is constructed to reduce the influence of weak observability state variables on the whole filter, which can improve the estimation accuracy of transfer alignment. Simulations and experiment tests of the airborne pod and the master strapdown inertial navigation systems show that the adaptive transfer alignment method based on the observability analysis can overcome the shortage of the weak observability state variables, so as to improve the alignment and the navigation performance in practical applications, thus improving the adaptability of the airborne pod.

## Introduction

**Figure 1:**
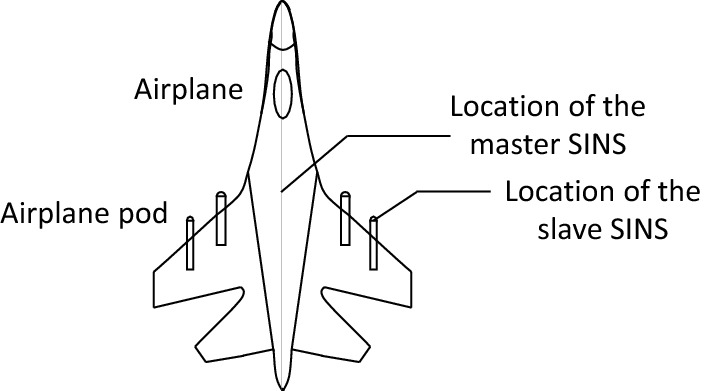
The schematic diagram of master SINS and slave SINS.

For advanced aircraft, fast and accurate initial alignment can not only improve the navigation accuracy of airborne strapdown inertial navigation system (SINS), but also improve the aircraft's response speed to enhance its survivability^1^. For the airborne pod strapdown inertial navigation system, because of its wide range of applications, it is necessary to use the host aircraft's inertial navigation system for the transfer alignment as quickly and accurately as possible in the flight process of the aircraft, so as to reduce the search range of the target and improve the acquisition efficiency^2,3^. The schematic diagram of master SINS and slave SINS is shown as Fig. 1.

However, airborne pod is mainly used in the combat in the complex environment, including many types of tasks such as detection, aiming and acquisition during the high dynamic flight process^4,5^. This situation puts forward high requirements for the rapidity, accuracy and reliability of airborne pod transfer alignment. In order to achieve high-precision transfer alignment of airborne pod, it is necessary to study the methods to improve the transfer alignment performance according to the characteristics of airborne pod^6^. Due to the limitation of cost and volume, the accuracy and stability of the strapdown inertial navigation system equipped on the airborne pod are usually lower than that of the airborne. Even after the ground calibration, there are still big errors in the actual flight process, which greatly affects the performance of the airborne pod transfer alignment^7,8^. Hence, it is necessary to consider the accuracy of the airborne pod and the environment of the flight process.

In recent years, the airborne transfer alignment methods have been still kept in research. The relative navigation method has been proposed, where the alignment process is achieved by computing the relative motion between different inertial units^9^. A non-linear relation between the flexural angle and flexural lever arm variation method is to establish a novel 27-state non-linear model of the transfer alignment^10^. The transfer alignment model based on fiber Bragg grating has been performed to solve the problem of the coupling movement error caused by flexible deformation^11^. Three rapid transfer alignment matching methods "velocity plus attitude", "velocity plus rate" and "attitude plus rate" have been compared from different aspects in this paper^12^. However, in the actual flight process of aircraft, due to the change of air environment, maneuvering impact and so on, the transfer alignment performance of airborne pod is uncertain. Different maneuvering modes and matching modes will affect the observability of the system, and then affect the accuracy of transfer alignment and stability of the system^13,14^. Due to these reasons, there are a large number of adaptive Kalman filtering methods, such as Adaptive Extended Kalman Filter (AEKF)^15^, variational Bayesian based AKF (VAKF)^16^ and so on^17–19^. However, the observability is not considered in these methods. Introducing the state estimation with poor observability into the system for error correction will lead to error accumulation, and may also bring coupling interference error to other observable state parameters, resulting in the decline of filtering accuracy^20,21^. So, the general transfer alignment methods may be no longer applicable due to these reasons.

In this paper, an adaptive transfer alignment method based on the observability analysis for airborne pod strapdown inertial navigation system is proposed. Through the observability analysis, the observability of each state variable is obtained. According to the weight of the observability, a transfer alignment filter algorithm based on adaptive adjustment factor has been constructed to reduce the influence of weak observability state variables on the whole filter, which can improve the estimation accuracy of transfer alignment. An experimental system has been built and different experiments have been designed to prove the effectiveness of the method.

## Influence of filter performance on transfer alignment performance

In the airborne pod transfer alignment process, due to the change of air environment and maneuvering impact, there are a lot of uncertainties in the system error equation and system noise. It is difficult to establish the error modeling. The traditional filter can't detect and adjust automatically, so it is limited in application.

On the other hand, the observability is the key problem of the alignment filter in estimating system state variables. The main task of transfer alignment is to estimate the system state variables and modify them in Strapdown inertial navigation system (SINS). When using Kalman filter to estimate the state variables, if the state variables of the system can be observed, the estimation error standard deviation (EESD) will gradually converge and reach a stable value^22,23^. When the system is not completely observable, the EESD of the unobservable state variables will not converge and be less than a certain boundary value^24^. In the transfer alignment, different maneuvering modes and matching modes will lead to the problem of whether the state vector can be observed. The observability of the system determines the convergence speed and accuracy of the state estimation, and then affects the accuracy of transfer alignment and the stability of the system. SINS needs to update its own state according to the filter estimation. ${\hat {\mathbf{x}}^k}$ represents the state estimate at k moment, and ${\hat {\mathbf{x}}^{k + {1}}}$ represents the state estimate at k + 1 moment, $\hat {\mathbf{x}}$ represents the state estimation correction value according to the measurement information. The update equation can be expressed as:

1 $${\hat {\mathbf{x}}^{k + 1}} = {\hat {\mathbf{x}}^k} + \hat {\mathbf{x}}$$

If the observability of the state parameter in the transfer alignment filter is poor, that is, the matching measurement information can not directly measure the state vector. If the poor observability state estimation is introduced into the system for error correction, it will lead to error accumulation, and may also bring coupling interference error to other observable state parameters. Therefore, after the transfer alignment matching mode is determined, it is necessary to analyze the observability of SINS and dynamically select the state parameters to improve the accuracy and stability of the filter.

## Method

According to the influence of uncertain noise and state observability on the transfer alignment performance of airborne SINS, it can be seen that the performance of filter will affect the accuracy and robustness of transfer alignment algorithm. Therefore, in order to further improve the accuracy and robustness of transfer alignment filtering, an adaptive transfer alignment filtering algorithm based on observability analysis is proposed in this section.

### Observability analysis

Observability can be measured by observability degree. The observability degree can reflect the inherent characteristics of transfer alignment model more precisely. At present, the commonly used observability analysis methods mainly include the eigenvalue method of estimating covariance matrix and the singular value decomposition (SVD) method of observability matrix^25^. The eigenvalue method for estimating the covariance matrix needs to obtain the covariance matrix of the estimated state error in the calculation process, and then the observability degree can be judged. SVD is the method that the singular value and singular vector of the observability matrix are used to quantitatively describe the observability of the model. Its biggest advantage is that it can get the analysis results of observability and observability at the same time.

**Figure 2:**
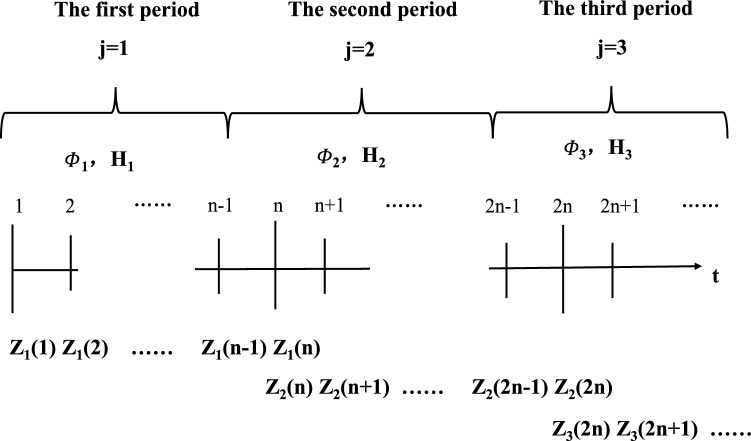
The discrete measurement sequence.

The specific steps of improved singular value decomposition method to analyze time-varying systems are as follows:

1. Select the first period of the time-varying system and make j = 1;
2. Calculate the observability matrix Q for the corresponding period;
3. Determine the current SOM matrix Q_S_;
4. The measurement Z in this period is calculated according to the accuracy and size of the observation used by the time-varying system;
5. The singular value of the observability matrix Q_S_ is calculated in the current period;
6. The size of the state variable corresponding to the each singular value has been calculated. According to the size, we can judge which state variables are observable or not;
7. If the current period is not the last period, we need analyses the second period. Let j = 2, return to step 2, and continue all of periods until the analysis is completed. The discrete measurement sequence is shown as Fig. 2.

Taking the discrete homogeneous linear system as an example, the system equation and observation equation can be expressed as:

2 $$\left\{ {\begin{array}{*{20}{c}} {{\mathbf{X}}(k + 1) = \Phi (k){\mathbf{X}}(k)} \\ {{\mathbf{Z}}(k) = {\mathbf{H}}(k){\mathbf{X}}(k)} \end{array}} \right.$$

Suppose there is a set of measurements that are ${\mathbf{Z}}(0),{\mathbf{Z}}(1), \ldots ,{\mathbf{Z}}(k)$, the initial state $X(0)$ can be expressed as a function of the measured value:

3 $$\begin{gathered} {\mathbf{Z}}(0) = {\mathbf{HX}}(0) \\ {\mathbf{Z}}(1) = {\mathbf{H}}\Phi (0){\mathbf{X}}(0) \\ {\mathbf{Z}}(2) = {\mathbf{H}}\Phi (1)\Phi (0){\mathbf{X}}(0) \\ \vdots \\ {\mathbf{Z}}(k) = {\mathbf{H}}\Phi (k - 1)\Phi (k - 2) \cdots \Phi (1)\Phi (0){\mathbf{X}}(0) \\ \end{gathered}$$

Let,

4 $$Q = \left[ {\begin{array}{*{20}{c}} {\mathbf{H}} \\ {{\mathbf{H}}\Phi (0)} \\ {{\mathbf{HF}}(1)\Phi (0)} \\ \vdots \\ {{\mathbf{H}}\Phi (k - 1)\Phi (k - 2) \cdots \Phi (1)\Phi (0){\mathbf{X}}(0)} \end{array}} \right],Z = \left[ {\begin{array}{*{20}{c}} {Z(0)} \\ {Z(1)} \\ {Z(2)} \\ \vdots \\ {Z(k)} \end{array}} \right]$$

So,

5 $${\mathbf{Z}} = {\mathbf{QX}}(0)$$

where, ${\mathbf{Q}}$ is the observability matrix of dynamic system. The estimation of initial state ${\mathbf{X}}(0)$ depends on the characteristics of observable matrix. The singular value decomposition of the matrix ${\mathbf{Q}}$ is used to solve the problem.

6 $${\mathbf{Q}} = {\mathbf{USV}}^T$$

where, $${\mathbf{U}} = [\begin{array}{*{20}{c}} {{{\mathbf{u}}_1}}&{{{\mathbf{u}}_2}}& \cdots &{{{\mathbf{u}}_m}} \end{array}]$$, $${\mathbf{V}} = [\begin{array}{*{20}{c}} {{{\mathbf{v}}_1}}&{{{\mathbf{v}}_2}}& \cdots &{{{\mathbf{v}}_n}} \end{array}]$$ are the orthogonal matrix, ${\mathbf{S}} = diag({\Lambda_{r \times r}},0)$ is the $m \times r$ matrix. Where, $${\Lambda_{r \times r}} = diag(\begin{array}{*{20}{c}} {\sigma_1}&{\sigma_2}&{...}&{\sigma_r} \end{array})$$ is the diagonal matrix, ${\sigma_1} \geqslant {\sigma_2} \geqslant \cdots \geqslant {\sigma_r} \geqslant 0$ is the singular value of the matrix ${\mathbf{Q}}$. So the equation can be described as:

7 $${\mathbf{Z}} = ({\mathbf{USV}}^T){\mathbf{X}}(0) = (\sum\limits_{i = 1}^r {{\sigma_i}{{\mathbf{u}}_i}{\mathbf{v}}_i^T} ){\mathbf{X}}(0) = \sum\limits_{i = 1}^r {{\sigma_i}({\mathbf{v}}_i^T{\mathbf{X}}(0)){{\mathbf{u}}_i}}$$

Obviously, the projection of the initial state ${\mathbf{X}}(0)$ on the tensor subspace is transformed into the observation ${\mathbf{Z}}$. Therefore, the state ${\mathbf{X}}(0)$ is uniquely determined with r measurements. If ${\sigma_i} > 0(i = 1,2, \ldots ,r)$, the initial state can be estimated and determined by using these m measurements ${\mathbf{Z}}$. So,

8 $${\mathbf{X}}(0) = {({\mathbf{USV}}^T)^{ - 1}}{\mathbf{Z}} = \sum\limits_{i = 1}^r {\left( {\frac{{{\mathbf{u}}_i^T{\mathbf{Z}}{v_i}}}{{\sigma_i}}} \right)}$$

Let $${\mathbf{V}} = \left[ {\begin{array}{*{20}{c}} {{\mathbf{V}}_1}&{{\mathbf{V}}_2} \end{array}} \right]$$, where, $${{\mathbf{V}}_1} = \left[ {\begin{array}{*{20}{c}} {{\mathbf{v}}_1}&{{\mathbf{v}}_2}& \cdots &{{\mathbf{v}}_l} \end{array}} \right]$$, $${{\mathbf{V}}_2} = \left[ {\begin{array}{*{20}{c}} {{{\mathbf{v}}_{l + 1}}}&{{{\mathbf{v}}_{l + 2}}}& \cdots &{{\mathbf{v}}_r} \end{array}} \right]$$. ${{\mathbf{V}}_2}$ represents the null space of a matrix ${\mathbf{Q}}$. In this case, the initial state ${\mathbf{X}}(0)$ can be expressed as:

9 $${\mathbf{X}}(0) = \sum\limits_{i = 1}^r {\left( {\frac{{{\mathbf{u}}_i^T{\mathbf{Z}}{v_i}}}{{\sigma_i}}} \right) + } \sum\limits_{i = l + 1}^{lr} {{\alpha_i}{{\mathbf{v}}_i}}$$

where, ${\alpha_i}(i = l + 1,l + 2, \ldots ,r)$ is an arbitrary coefficient in the null space. It is obvious that there are many possible solutions to this coefficient. For the singular value less than a fixed value, it may cause multiple ${\mathbf{X}}(0)$ singularities. When evaluating the influence of different vehicle maneuvering modes on the estimation accuracy of initial state ${\mathbf{X}}(0)$, the internal relationship between singular value and initial state should be studied. When the observation has a constant norm, the initial state ${\mathbf{X}}(0)$ forms an ellipsoid, the equation is:

10 $$\sum\limits_{i = 1}^r {{{\left( {\frac{{{\mathbf{v}}_i^T{\mathbf{X}}(0){{\mathbf{u}}_i}}}{{\alpha_i}}} \right)}^2}} = {\left| {\mathbf{Z}} \right|^2}$$

where, ${\alpha_i} = 1/{\sigma_i}$ represents the length of the main axis of the ellipsoid. Obviously, the volume of the ellipsoid is determined by singular value. When the singular value is large, the volume is small and ${\mathbf{X}}(0)$ is small. Therefore, the upper boundary is expressed as:

11 $${\mathbf{X}}(0) \leqslant \frac{\left| {\mathbf{Z}} \right|}{{\sigma_r}}$$

If ${\sigma_r}$ is larger, ${\mathbf{X}}(0)$ will be small; When ${\sigma_r}$ is 0, the estimation problem becomes a singular problem. Because the estimation is boundless, that is, ${\mathbf{X}}(0)$ cannot be determined by the measurement ${\mathbf{Z}}$. The observability degree is defined as the ratio of the singular value, namely:

12 $${\eta_i} = \frac{{\sigma_i}}{{\sigma_0}}$$

where, ${\eta_i}$ is the observability degree of the *i*th state^26^. ${\sigma_i}$ is the singular value corresponding to the state. ${\sigma_0}$ is the singular value corresponding to the state with the measurement.

### Adaptive transfer alignment algorithm based on based on observability analysis

There are many adaptive algorithms in current research, such as Sage Husa adaptive algorithm, Kalman filter algorithm with attenuation factor. The former method has higher accuracy, but it is difficult to guarantee the stability of the system with higher order. The latter is simple in structure, but it reduces the filtering accuracy. In this section, Sage Husa adaptive algorithm has been adopted to improve its filtering performance. The statistical characteristics of unknown noise are obtained in real time based on the observed value ${{\mathbf{Z}}_k}$. The fading factor is introduced to adjust the filtering gain matrix in real time according to the observability degree of state variables.

In Sage Husa adaptive algorithm, the filtering equation is shown as follows:

13 $$\left\{ {\begin{array}{*{20}{l}} {{{\hat {\mathbf{X}}}_{k/k - 1}} = {\Phi_{k,k - 1}}{{\hat {\mathbf{X}}}_{k - 1}} + {{\hat {\mathbf{q}}}_{k - 1}}} \\ {{{\mathbf{P}}_{k/k - 1}} = {\Phi_{k,k - 1}}{{\mathbf{P}}_{k - 1}}\Phi_{k,k - 1}^T + {{\hat {\mathbf{Q}}}_{k - 1}}} \\ {{{\mathbf{K}}_k} = {{\mathbf{P}}_{k/k - 1}}{\mathbf{H}}_k^T{{({{\mathbf{H}}_k}{{\mathbf{P}}_{k/k - 1}}{\mathbf{H}}_k^T + {{\hat {\mathbf{R}}}_k})}^{ - 1}}} \\ {{{\mathbf{e}}_k} = {{\mathbf{Z}}_k} - {{\mathbf{H}}_k}{{\hat {\mathbf{X}}}_{k/k - 1}} - {{\hat {\mathbf{r}}}_{k - 1}}} \\ {{{\hat {\mathbf{X}}}_k} = {{\hat {\mathbf{X}}}_{k/k - 1}} + {{\mathbf{K}}_k}{e_k}} \\ {{{\mathbf{P}}_k} = (I - {{\mathbf{K}}_k}{{\mathbf{H}}_k}){{\mathbf{P}}_{k/k - 1}}} \end{array}} \right.$$

The time varying noise estimator is:

14 $$\left\{ {\begin{array}{*{20}{l}} {{{\hat {\mathbf{r}}}_k} = (1 - {d_k}){{\hat {\mathbf{r}}}_{k - 1}} + {d_k}({{\mathbf{Z}}_k} - {{\mathbf{H}}_k}{{\hat {\mathbf{X}}}_{k/k - 1}})} \\ {{{\hat {\mathbf{R}}}_k} = (1 - {d_k}){{\hat {\mathbf{R}}}_{k - 1}} + {d_k}({{\mathbf{e}}_k}{\mathbf{e}}_k^T - {{\mathbf{H}}_k}{{\mathbf{P}}_{k/k - 1}}{\mathbf{H}}_k^T)} \\ {{{\hat {\mathbf{q}}}_k} = (1 - {d_k}){{\hat {\mathbf{q}}}_{k - 1}} + {d_k}({{\hat {\mathbf{X}}}_k} - {\Phi_{k,k - 1}}{{\hat {\mathbf{X}}}_{k - 1}})} \\ {{{\hat {\mathbf{Q}}}_k} = (1 - {d_k}){{\hat {\mathbf{Q}}}_{k - 1}} + {d_k}({{\mathbf{K}}_k}{e_k}e_k^T{\mathbf{K}}_k^T + {{\mathbf{P}}_k} - {\Phi_{k,k - 1}}{{\mathbf{P}}_{k - 1}}\Phi_{k,k - 1}^T)} \end{array}} \right.$$

where, ${d_k} = {{\left( {1 - b} \right)} \mathord{\left/ {\vphantom {{\left( {1 - b} \right)} {\left( {1 - {b^{k + 1}}} \right)}}} \right. \kern-\nulldelimiterspace} {\left( {1 - {b^{k + 1}}} \right)}},0 < b < {1}$. $b$ is the forgetting factor, which can limit the memory length of the filter. ${{\mathbf{H}}_k}$ is the measurement matrix. ${{\mathbf{P}}_{k/k - 1}}$ is the covariance matrix. ${{\mathbf{K}}_k}$ is the gain matrix. ${{\mathbf{Q}}_k}$ is the variance matrix of system noise. ${{\mathbf{Z}}_k}$ is the measurement. ${{\mathbf{R}}_k}$ is the variance matrix of measurement noise. ${\Phi_{k,k - 1}}$ is the state transition matrix from k to k + 1 moment. ${e_k}$ is the measurement residual of filter. ${{\mathbf{q}}_k}$ is the mean of system noise and ${{\mathbf{r}}_k}$ is the mean of measurement noise.

However, this method obviously increases the calculation of the statistical characteristics of the system noise, which makes the calculation and filtering more complex. The real-time performance becomes worse, and the engineering implementation becomes more difficult. In order to improve the filtering calculation efficiency of the transfer alignment, it is generally considered that the system noise in the navigation system is stable. That is, only the measurement noise ${\hat {\mathbf{R}}_k}$ is estimated because it has a significant effect on filtering. At this time, Sage Husa algorithm becomes a simplified sage Husa adaptive filtering algorithm^27^. This algorithm is relatively simple and practical in engineering. Assuming ${\hat {\mathbf{r}}_k} = 0$, ${\hat {\mathbf{q}}_k} = 0$ and ${{\mathbf{Q}}_k}$ is constant, the simplified Sage Husa filtering algorithm is shown as follows:

15 $$\left\{ {\begin{array}{*{20}{l}} {{d_k} = {{\left( {1 - b} \right)} \mathord{\left/ {\vphantom {{\left( {1 - b} \right)} {\left( {1 - {b^{k + 1}}} \right)}}} \right. \kern-\nulldelimiterspace} {\left( {1 - {b^{k + 1}}} \right)}}} \\ {{{\hat {\mathbf{X}}}_{k/k - 1}} = {\Phi_{k,k - 1}}{{\hat {\mathbf{X}}}_{k - 1}}} \\ {{{\mathbf{P}}_{k/k - 1}} = {\Phi_{k,k - 1}}{{\mathbf{P}}_{k - 1}}\Phi_{k,k - 1}^T + {{\mathbf{Q}}_{k - 1}}} \\ {{{\mathbf{e}}_k} = {{\mathbf{Z}}_k} - {{\mathbf{H}}_k}{{\hat {\mathbf{X}}}_{k/k - 1}}} \\ {{{\mathbf{K}}_k} = {{\mathbf{P}}_{k/k - 1}}{\mathbf{H}}_k^T{{({{\mathbf{H}}_k}{{\mathbf{P}}_{k/k - 1}}{\mathbf{H}}_k^T + {{\hat {\mathbf{R}}}_k})}^{ - 1}}} \\ {{{\hat {\mathbf{X}}}_k} = {{\hat {\mathbf{X}}}_{k/k - 1}} + {{\mathbf{K}}_k}{e_k}} \\ {{{\mathbf{P}}_k} = (I - {{\mathbf{K}}_k}{{\mathbf{H}}_k}){{\mathbf{P}}_{k/k - 1}}} \end{array}} \right.$$

If the observability of a state parameter of the transfer alignment filter is poor, the introduction of the state vector with poor observability into the filter will lead to poor filtering accuracy, and may bring coupling interference error to other observable state parameters. Therefore, in order to solve the problem of the poor observability, a fading factor ${\lambda_k}$ is introduced into the covariance matrix of state prediction error. The fading factor can be adjusted according to the weight of observability of each state variable, so as to adjust the filter gain matrix ${{\mathbf{K}}_k}$.

${\lambda_k}$ is the time-varying fading factor matrix. It is defined as follows:

16 $${\lambda_k} = \left[ {\begin{array}{*{20}{c}} {\lambda_1}&0& \cdots &0 \\ 0&{\lambda_2}& \cdots &0 \\ \vdots & \vdots &{}& \vdots \\ 0&0& \cdots &{\lambda_n} \end{array}} \right]$$

where, $n$ is the dimension of the state quantity, $\lambda_k^i(i = 1,2, \ldots ,n)$ is the fading factor corresponding to each state. The fading factor can be calculated by the following steps:

Step 1: Set the initial value $\lambda_k^i$, $\lambda_1^1 = 1$;

Step 2: Get the geometric mean $\gamma$ of the observability ${\eta_i}$. Geometric mean (GM) is the n-th root of the product of observations, which is used to calculate ratio or dynamic mean.

Step 3: The adaptive factor ${\lambda_k}$ is obtained according to the geometric average of the second step.

17 $$\lambda {(i,i)_{k + 1}} = {\left( {\sqrt {{\eta_i}/\gamma } } \right)^\frac{1}{n}}\lambda {(i,i)_k},\;(i = 1,2,...,n)$$

Because the adaptive factor is directly related to the observability, the larger observability is, the larger the weight is. The fading factor is introduced to adjust the gain matrix ${K_k}$ on-line in real time so that it is related to the observability, and the prediction covariance matrix is calculated according to ${\lambda_k}$.

18 $${{\mathbf{P}}_{k + 1/k}} = {\lambda_{k + 1}}{{{\varvec{\Phi}}}_{k + 1/k}}{{\mathbf{P}}_{k/k}}{{{\varvec{\Phi}}}^T}_{k + 1/k} + {{\mathbf{T}}_k}{{\mathbf{Q}}_{k + 1}}{{\mathbf{T}}^T}_k$$

${{\mathbf{T}}_k}$ is the system noise driving matrix. According to the above equation, the gain matrix of the system is obtained as follows:

19 $${{\mathbf{K}}_{k + 1}} = {{\mathbf{P}}_{k + 1/k}}{{\mathbf{H}}^T}_{k + 1}{\left[ {{{\mathbf{H}}_{k + 1}}{{\mathbf{P}}_{k + 1/k}}{{\mathbf{H}}^T}_{k + 1} + {{\mathbf{R}}_{k + 1}}} \right]^{ - 1}}$$

It can be seen from above equations that method of the adaptive filtering based on observability introduces fading factor ${\lambda_k}$, which plays an important role in the calculation of one-step prediction covariance matrix. When the observability of the system state changes, it increases accordingly, and the state error with large observability is enhanced. At the same time, the filter can estimate and modify the statistical rules and characteristics of measurement noise online, so as to reduce the error of state estimation and improve the alignment accuracy and rapidity of the system.

## Establishment of transfer alignment model

Because the delay of the reference navigation information has a great influence on velocity and attitude, the velocity/attitude matching method is selected for alignment, and the time delay is estimated and compensated. The state variables of transfer alignment include sub INS misalignment angle $${\phi^n} = {[\begin{array}{*{20}{c}} {\phi_E}&{\phi_N}&{\phi_U} \end{array}]^T}$$, velocity error $$\delta {{\mathbf{V}}^n} = {\left[ {\begin{array}{*{20}{c}} {\delta {V_E}}&{\delta {V_N}}&{\delta {V_U}} \end{array}} \right]^T}$$, acceleration bias $$\nabla^{bs} = {[\begin{array}{*{20}{c}} {\nabla_x^{bs}}&{\nabla_y^{bs}}&{\nabla_z^{bs}} \end{array}]^T}$$, gyro drift $$\varepsilon^{bs} = {[\begin{array}{*{20}{c}} {\varepsilon_x^{bs}}&{\varepsilon_y^{bs}}&{\varepsilon_z^{bs}} \end{array}]^T}$$, installation error angle $$\mu = {[\begin{array}{*{20}{c}} {\mu_x}&{\mu_y}&{\mu_z} \end{array}]^T}$$ and delay time $\Delta t$. Namely:

20 $${\mathbf{X}}(t) = {\left[ {\begin{array}{*{20}{l}} {\phi^n}&{\delta {{\mathbf{V}}^n}}&{\varepsilon^{bs}}&{\nabla^{bs}}&\mu &{\Delta t} \end{array}} \right]^T}$$

The state error equation of strapdown inertial navigation system is as follows:

21 $$\left\{ {\begin{array}{*{20}{l}} {{{\dot \phi }^n} = - \omega_{in}^n \times {\phi^n} + \delta \omega_{in}^n + C_{bs}^n\varepsilon^{bs}} \\ {\delta {{\dot \nu }^n} = {f^n} \times {\phi^n} - {(2}\delta \omega_{ie}^n + \delta \omega_{en}^n{)} \times {v^n} - {(}2\omega_{ie}^n + \omega_{en}^n{)} \times \delta {v^n} + C_{bs}^n{\nabla^{bs}}} \\ {{{\dot \varepsilon }^{bs}} = {0,}{{\dot \nabla }^{bs}} = {0}} \\ {\dot \mu = 0} \end{array}} \right.$$

where, $\omega_{in}^n$ is the angular velocity of the navigation system relative to the inertial system obtained by the sub inertial navigation system, and $\delta \omega_{in}^n$ is the error of angular velocity $\omega_{in}^n$; $C_{bs}^n$ is the attitude matrix of the sub ins carrier system relative to the navigation system; ${f^n}$ is the projection of the specific force of the sub ins in the navigation system; $\omega_{ie}^n$ is the projection of the earth rotation rate measured by the sub INS on the navigation system, and $\delta \omega_{ie}^n$ is the error of angular velocity $\omega_{ie}^n$; $\omega_{en}^n$ is the projection of the angular rate of the navigation coordinate relative to the earth coordinate in the navigation coordinate, and $\delta \omega_{en}^n$ is the error of angular velocity $\omega_{en}^n$.

The delay time Δ*t* in the transfer alignment is usually treated as a random constant. So the model of the delay time $\Delta t$ is as follows:

22 $$\Delta \dot t = 0$$

For the velocity/attitude matching mode, the observation ${\mathbf{Z}}(t)$ in the alignment filter is chosen as the velocity and attitude difference between the main and sub INS. The velocity measurement ${\mathbf{Z}}^{velo}$ is shown as:

23 $${\mathbf{Z}}^{velo} = {\mathbf{V}}_s^n - {\mathbf{V}}_m^n = \delta {{\mathbf{V}}^n} + \left[ {\begin{array}{*{20}{c}} {{{\dot V}_E}} \\ {{{\dot V}_N}} \\ {{{\dot V}_U}} \end{array}} \right]\Delta t$$

The attitude measurement ${\mathbf{Z}}^{atti}$ is acquired by using ${\mathbf{Z}}_{bs}^{atti}$:

24 $${\mathbf{Z}}_{bs}^{atti} = \hat {\mathbf{C}}_{bs}^n\hat {\mathbf{C}}_{bm}^{bs}{\mathbf{C}}_n^{bm}$$

where, ${\mathbf{C}}_n^{bm}$ is the direction cosine matrix from the reference inertial navigation coordinate system to the navigation coordinate system. $\hat {\mathbf{C}}_{bm}^{bs}$ is the direction cosine matrix from reference inertial coordinate system to sub inertial coordinate system, which contains calculation error. $\hat {\mathbf{C}}_{bs}^n$ is the direction cosine matrix from the sub inertial navigation carrier coordinate system to the navigation coordinate system, which also contains the calculation error.

25 $$\left\{ {\begin{array}{*{20}{c}} {\hat {\mathbf{C}}_n^{bs} = [I - (\phi \times )]{\mathbf{C}}_n^{bs} = {\mathbf{C}}_n^{bs} - (\phi \times ){\mathbf{C}}_n^{bs}} \\ {\hat {\mathbf{C}}_{bm}^{bs} = [I - (\mu \times )]{\mathbf{C}}_{bm}^{bs} = {\mathbf{C}}_{bm}^{bs} - (\mu \times ){\mathbf{C}}_{bm}^{bs}} \end{array}} \right.$$

where, $\phi$ is the attitude misalignment angle between the calculated navigation coordinate system and the real navigation coordinate system of the sub INS. $\mu$ is the installation error angle from the reference inertial navigation coordinate system to the sub inertial navigation coordinate system. Both of them are small angles.

26 $$\begin{aligned} {\mathbf{Z}}_{bs}^{atti} = & \hat {\mathbf{C}}_{bs}^n\hat {\mathbf{C}}_{bm}^{bs}{\mathbf{C}}_n^{bm} \\ = & [{\mathbf{C}}_n^{bs} - (\phi \times ){\mathbf{C}}_n^{bs}][{\mathbf{C}}_n^{bs} - (\mu \times ){\mathbf{C}}_n^{bs}] \\ = & I - (\phi \times ) - {\mathbf{C}}_{bs}^n(\mu \times ){\mathbf{C}}_n^{bs} + (\phi \times ){\mathbf{C}}_{bs}^n(\mu \times ){\mathbf{C}}_n^{bs} \\ \end{aligned}$$

Taking into account that $\phi$ and $\mu$ are small angles, the above equation can be simplified as:

27 $${\mathbf{Z}}_{bs}^{atti} = {\mathbf{I}} - (\phi \times ) - {\mathbf{C}}_{bs}^n(\mu \times ){\mathbf{C}}_n^{bs} = {\mathbf{I}} - (\phi \times ) - [({\mathbf{C}}_{bs}^n\mu ) \times ]$$

The velocity measurement ${\mathbf{Z}}^{atti}$ is shown as:

28 $${\mathbf{Z}}^{atti} = \left[ {\begin{array}{*{20}{c}} {{\mathbf{Z}}_{bs}^{atti}(2,3)} \\ {{\mathbf{Z}}_{bs}^{atti}(3,1)} \\ {{\mathbf{Z}}_{bs}^{atti}(1,2)} \end{array}} \right] = \phi + {\mathbf{C}}_{bs}^n\mu + {{\mathbf{v}}_\phi } + \left[ {\begin{array}{*{20}{c}} {{{\dot \phi }_x}} \\ {{{\dot \phi }_y}} \\ {{{\dot \phi }_z}} \end{array}} \right]\Delta t$$

where, ${{\mathbf{v}}_\phi }$ is the attitude measurement noise.

## Simulation and experiment results

In this section, simulations and experiments have been accomplished to verify and evaluate the performance of the proposed method. Firstly, the observability of the system under different maneuvering modes is analyzed. Secondly, the simulation performance of the adaptive transfer alignment algorithm based on observability has been compared with that of the traditional transfer alignment algorithm. Thirdly, a vehicle experiment has been verified the proposed method is superior to the traditional method in the practical use.

### Simulation analysis

In order to verify the estimation effect of the adaptive transfer alignment algorithm based on observability analysis, the transfer alignment of airborne SINS is simulated in this section.

(1) The observability of the system under different maneuvering modes is analyzed.

**Figure 3:**
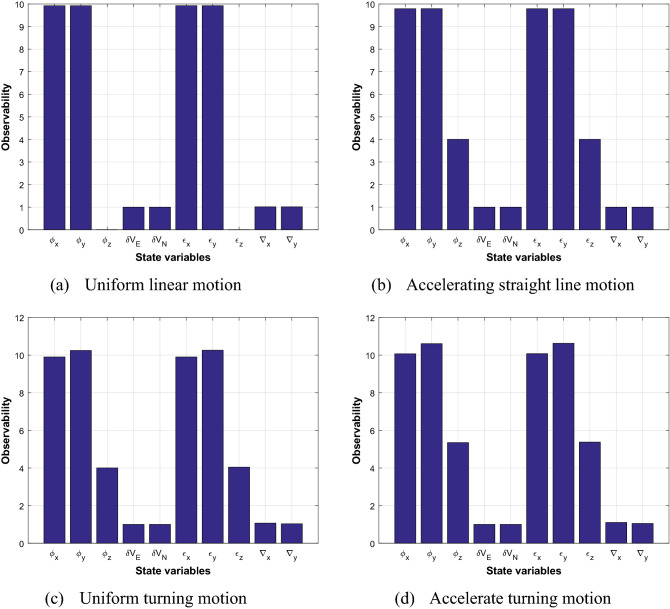
Comparison of state observability degree under 4 different maneuvering modes.

The transfer alignment is simulated in velocity matching mode. The simulated flight path includes 4 different maneuvering modes: uniform linear motion, accelerated linear motion, uniform turning motion and accelerated turning motion. The method based on singular value decomposition is used to analyze the observability of state variables. Figure 3 compares the observability degree of the state variables under 4 different maneuvering modes.

It can be seen from Fig. 3 that since the velocity information is used as the measurement for matching, the observability of velocity variable is constant and it is defined as 1 according to the definition of observability. In addition, the observability of other state variables is different under the 4 maneuvering modes. In the uniform linear motion, z-axis misalignment angle *φz* and z-axis gyroscope drift *ϵz* are not observable, but the observability increases significantly after changing to acceleration or turning maneuver.

(2) The performance of the proposed method is compared with the traditional method.

The simulation conditions are set as follows: the initial longitude, latitude and altitude of the designed aircraft are 118°, 32° and 1500 m respectively, the initial attitude errors are 0.2°, 0.1° and 0.1° respectively, the total time is 300 s. The settings of the reference inertial navigation system and the airborne pod inertial navigation system are shown in Table 1.

**Table 1:** Parameters of navigation sensors.

Sensor	Error sources	Parameters value	Relevant time
Reference inertial navigation system	Gyroscope constant drift	0.1°*/h*	0
	Gyroscope first order Markov process drift	0.1°*/h*	3600* s*
	Gyroscope white noise measurement	0.1°*/h*	0
	Accelerometer constant bias	1 × 10^-4^* g*	0
	Accelerometer first order Markov process	1 × 10^-4^* g*	1800*s*
Airborne pod inertial navigation system	Gyroscope constant drift	10°*/h*	0
	Gyroscope first order Markov process drift	10°*/h*	3600* s*
	Gyroscope white noise measurement	10°*/h*	0
	Accelerometer constant bias	3 × 10^-4^* g*	0
	Accelerometer first order Markov process	3 × 10^-4^* g*	1800*s*

**Figure 4:**
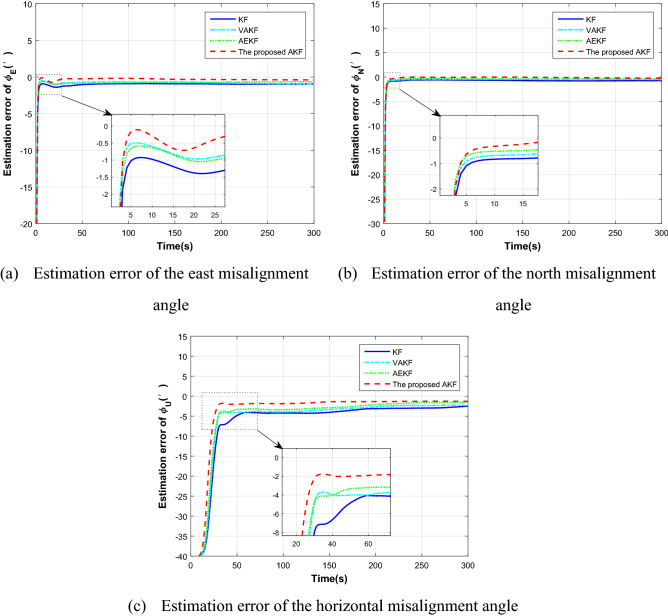
Misalignment angle estimation comparison.

The duration of the simulated flight path is 300 s. The aircraft accelerates at 3 m/s^2^ at the initial speed of 200 m/s in the first 30 s before sailing, and then flies in a straight line at a constant speed. The proposed AKF method is used for simulation verification, and KF, VAKF, AEKF algorithms mentioned in the introduction are used for comparison. Figure 4 shows the estimation results of different algorithms. The dotted line represents the proposed method, and the solid line represents the traditional transfer alignment method.

It can be seen from the figure that in the 300 s of the transfer alignment process, the proposed method has improved the estimation accuracy of misalignment angle compared with the traditional algorithm. The estimation accuracy of horizontal misalignment angle is improved by 0.5 arc minutes and azimuth misalignment angle is improved by 2 arc minutes. In terms of the convergence rate of the estimation curve, the proposed method can also quickly converge to the steady state. This is because the observability of the state variables is considered in the proposed algorithm. For the state variables with weak observability, the adaptive filtering algorithm can adjust the weight by fading adaptive factor, and maintain good state estimation ability, so as to improve the accuracy and rapidity of transfer alignment.

(3) When the noise of the sensor measurement is abnormal, the performance proposed method is compared with the traditional transfer alignment method.

**Figure 5:**
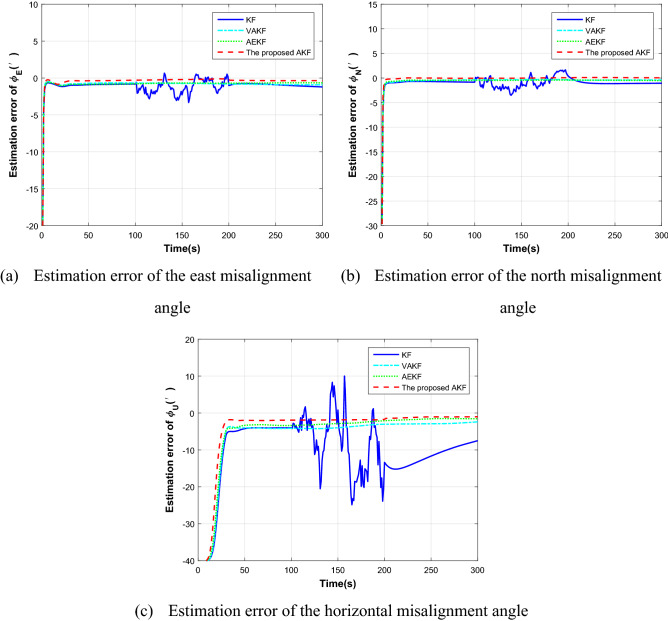
Misalignment angle estimation comparison with abnormal noise measurement.

In order to verify the adaptability of the proposed method, the simulation sets the measurement noise change during 100 ~ 200 s. Because the speed matching is used as the measurement information in this section, the standard deviation of speed measurement can be set from 0.1 to 1 m/s. Figure 5 shows the error angle estimation results of four different algorithms with abnormal noise measurement.

From Fig. 5, it can be seen that when the speed measurement noise is set in the period of 100 ~ 200 s, the measurement characteristics of the system will change, and the traditional algorithm can not change the parameters to adapt to the change of the noise measurement, resulting in the rapid deterioration of the transfer alignment accuracy; However, because the proposed algorithm can estimate and correct the measurement noise online by using the time-varying noise estimator. As a result, the adaptive transfer alignment algorithm still has good accuracy, so as to improve the accuracy and rapidity of transfer alignment.

### Ground experiment and discussion

**Figure 6:**
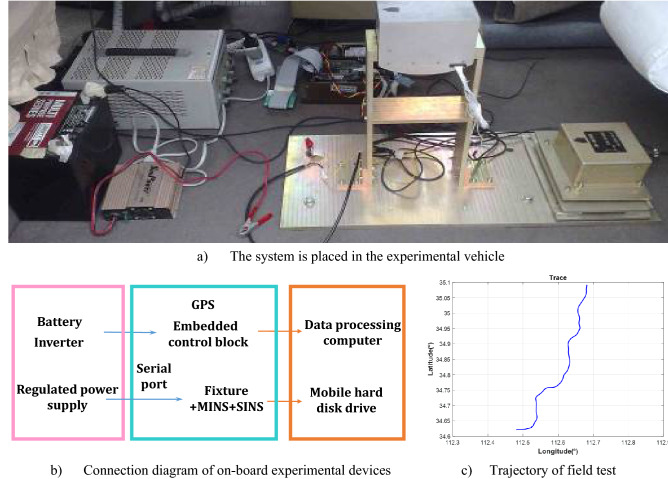
The vehicle experiment test platform and the tested trajectory.

To further verify the method proposed in the paper, the vehicle experiment platform has been constructed, as shown in Fig. 6a. We can see the connection diagram of on-board experimental devices from Fig. 6b. The performance of the algorithm has been analyzed and verified. The test platform contain Kalman’ the reference navigation system and the airborne pod navigation system (SINS). The reference navigation system can provide the reference information for the alignment. The equivalent gyroscope drift of the master INS is 0.5°/h, and the equivalent accelerometer bias is 100 μg. The gyroscope constant and random drift of sub INS is 5°/h, while accelerometer constant and random bias is 300 μg. The Rauch-Tung-Striebel post-processing smoothing algorithm is adopted to acquire the reference navigation solutions, which is often used to assess the performance of the alignment.

**Figure 7:**
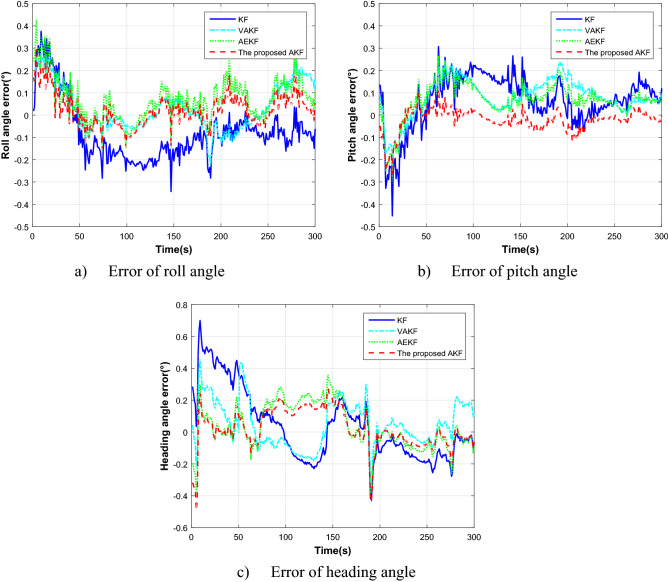
The alignment estimation error comparison in the accelerated linear motion.

The experiment was carried out in Henan Luoyang, the position of which is 112°3′ E, 34°3′ N. The alignment data of 300 s has been selected for verification. Figure 6c shows the trajectory of field test, where the start and end points are indicated. We select two segments of experiment data to verify the performance. Figure 7 and Table 2 show the alignment results with different methods in the accelerated linear motion.

**Table 2:** Results comparison in the accelerated linear motion.

Errors (degree)	KF		VAKF		AEKF		Proposed AKF	
	Average	SD	Average	SD	Average	SD	Average	SD
Roll angle	-0.079	0.134	0.032	0.104	0.071	0.097	0.024	0.075
Pitch angle	0.223	0.118	0.811	0.085	0.067	0.081	-0.066	0.052
Heading angle	0.034	0.221	0.048	0.145	0.036	0.143	0.021	0.124

**Figure 8:**
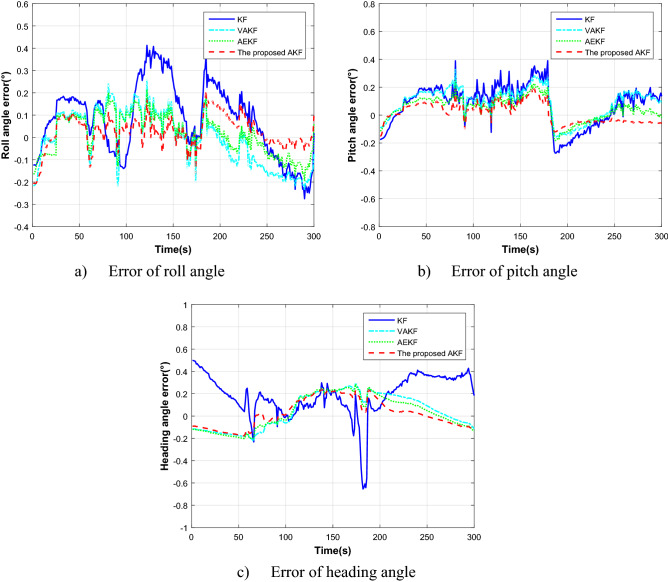
The alignment estimation error comparison in the turning motion.

Figure 8 and Table 3 show the alignment results with different methods in the turning motion.

**Table 3:** Results comparison in the turning motion.

Errors (degree)	KF		VAKF		AEKF		Proposed AKF	
	Average	SD	Average	SD	Average	SD	Average	SD
Roll angle	0.080	0.161	-0.004	0.124	0.015	0.092	0.024	0.073
Pitch angle	0.076	0.146	0.089	0.111	0.046	0.085	0.014	0.074
Heading angle	0.185	0.19	0.044	0.154	0.033	0.15	0.023	0.126

In conclusion, compared with other algorithms, the proposed AKF algorithm based on the observability analysis can improve the accuracy for the airborne pod INS. Especially, the proposed method is more robust in the practical use. With the help of the proposed adaptive filter where the predicted estimate covariance and measurement noise covariance matrix are adaptive adjusted, the results have better accuracy and shorter convergence time than the conventional method. The proposed method reduces the heading angle error to about 61.8% and the horizontal error to about 29.6% in the accelerated linear motion. The proposed method reduces the heading angle error to about 12.4% and the horizontal error to about 18.4% in the turning motion. It can generate the adaptive ability, so as to maintain a good navigation performance after the transfer alignment for the airborne pod INS.

## Conclusions

In this paper, according to the observability analysis method of system model, an adaptive transfer alignment method based on the observability analysis for airborne pod strapdown inertial navigation system has been proposed. With the weight of the observability, an adaptive adjustment factor is constructed to reduce the influence of the weak observability state variables on the system. At the same time, the time-varying noise estimator can be used to estimate and correct the measurement noise online, so as to comprehensively improve the accuracy and rapidity of the transfer alignment of the airborne strapdown inertial navigation system. Simulations and experiment tests of the airborne pod and the master strapdown inertial navigation systems show that the adaptive transfer alignment method based on the observability can overcome the shortage of the weak observability state variables. The system accuracy has been greatly improved in the practical use. In conclusion, the adaptive transfer alignment method based on the observability analysis can improve the transfer alignment and the navigation performance in practical applications, thus improving the adaptability of the airborne pod.
