# Author Correction: Adaptive transfer alignment method based on the observability analysis for airborne pod strapdown inertial navigation system

**DOI:** 10.1038/s41598-022-09223-8

**Published:** 2022-03-23

**Authors:** Weina Chen, Zhong Yang, Shanshan Gu, Yizhi Wang, Yujuan Tang

**Affiliations:** College of Intelligent Science and Control Engineering, Jinling Institute of Technology, Nanjing, China

Correction to: *Scientific Reports* 10.1038/s41598-021-04732-4, published online 18 January 2022

In the original version of this Article, Zhong Yang was omitted as a corresponding author. Correspondence and requests for materials should also be addressed to yz@jit.edu.cn.

The original Article has been corrected.

